# Associations of e-cigarette advertising exposure with curiosity and susceptibility among U.S. adolescents: National Youth Tobacco Surveys, 2014-2020

**DOI:** 10.1371/journal.pone.0303903

**Published:** 2024-09-20

**Authors:** Haijing Ma, Seth M. Noar, Kurt M. Ribisl

**Affiliations:** 1 College of Liberal Arts and Social Sciences, University of Houston-Victoria, Victoria, TX, United States of America; 2 Hussman School of Journalism and Media, University of North Carolina, Chapel Hill, NC, United States of America; 3 Department of Health Behavior, Gillings School of Global Public Health, University of North Carolina, Chapel Hill, NC, United States of America; Brazilian National Cancer Institute, BRAZIL

## Abstract

**Introduction:**

Despite an evolving e-cigarette environment, few studies have looked at adolescent exposure to e-cigarette advertising over time and its associations with curiosity about and susceptibility to using e-cigarettes. We examined e-cigarette advertising exposure and its associations with curiosity and susceptibility across multiple years among adolescents who have never used e-cigarettes.

**Methods:**

We obtained data from the National Youth Tobacco Surveys (NYTSs), 2014-2020 (N = 97,496). The NYTS identified e-cigarette advertising exposure from four channels: Internet, newspapers and magazines, convenience stores, and TV. Logistic regressions explored e-cigarette advertising exposure over time and the associations between exposure from the four channels and both curiosity and susceptibility to using e-cigarettes.

**Results:**

Youth exposure to e-cigarette advertising on the Internet and in convenience stores formed an increase-decrease-increase pattern from 2014 to 2020, whereas exposure in newspapers and magazines and on TV generally decreased over this period. Exposure on the Internet and in convenience stores was consistently associated with curiosity and susceptibility; but exposure in newspapers and magazines and on TV was sporadically associated with the outcomes.

**Conclusions:**

Despite a changing e-cigarette marketplace, youth were consistently exposed to e-cigarette advertising, especially on the Internet and in convenience stores. This pattern is worrisome, as it may increase youth curiosity and susceptibility to using e-cigarettes. Comprehensive tobacco prevention efforts to prevent e-cigarette use in adolescents should continue to restrict e-cigarette advertising and marketing, thereby reducing exposure and discouraging e-cigarette use. Regular efforts should also be made to educate adolescents about the risks of using e-cigarettes to counteract the impact of high e-cigarette advertising exposure.

## Introduction

According to the National Youth Tobacco Survey, 3.3% of middle school students and 14.1% of high school students in the U.S. reported current use of electronic cigarettes (i.e., e-cigarettes) in 2022, totaling 2.5 million middle and high school students [1]. Most e-cigarettes contain nicotine, which harms adolescent brain development [2]. E-cigarette vapor also emits other substances that may harm the lungs and lead to respiratory diseases [3–6]. Therefore, reducing e-cigarette use in adolescents is a priority for youth tobacco use prevention.

Evidence from national surveys has shown that curiosity about e-cigarettes is a primary reason behind adolescent use, and susceptibility to use is a predictor of future use [7, 8]. For example, 25.8% of youth who are never users of e-cigarettes reported being curious about e-cigarettes [9]. A longitudinal study revealed that e-cigarette susceptibility predicted subsequent initiation and past 30-day e-cigarette use 6 months later [10]. Exposure to e-cigarette advertising has been found to drive both curiosity and susceptibility [11–13]. U.S. adolescents who were exposed (vs. not) to e-cigarette advertising reported being more curious about e-cigarettes [13] and had greater susceptibility to using e-cigarettes [14].

E-cigarette companies have increased advertising expenditures in recent years and advertised e-cigarettes on multiple channels, such as the Internet and convenience stores, both of which are popular with adolescents [15, 16]. For instance, e-cigarette advertising spending increased from $6.4 million in 2011 to $115 million in 2014 [15]; as of 2018, annual e-cigarette advertising expenditures stood at $110 million [17]. As a result, adolescents are widely exposed to e-cigarette advertising: In 2016, about 78.2% of U.S. youth reported having seen at least one ad for e-cigarettes, compared to 68.9% in 2014 [18]. JUUL, which was one of the leading e-cigarette brands in the U.S. for youth and overall for many years, spent millions on advertising its products in wide-reaching channels [19]. However, JUUL halted all its broadcast, print, and digital product advertising in 2019, as it was being investigated by the Federal Trade Commission and was sued by several states for marketing their products to underage youth [20, 21]. Puff Bar, currently a popular youth e-cigarette brand, still widely advertises its products across multiple channels [22].

In response to rising youth use, federal, state, and local governments have also regulated e-cigarettes. For instance, in 2016, the US Food and Drug Administration released a Deeming Rule to assert their jurisdiction over the manufacturing, distribution, and marketing of e-cigarettes and issued warnings for unauthorized marketing of e-cigarettes [23]. The Federal Trade Commission developed guidelines on disclosing relationships between brands and influencers on social media marketing [24]. In addition, tribal, state, and local governments have taken additional, more stringent actions regarding the sale of e-cigarettes [25]. Some social media platforms such as Instagram also banned paid influencers to promote vaping [26]. However, there are no bans or comprehensive regulations on e-cigarette marketing and advertising in the US.

Despite the evolving e-cigarette advertising environment, it remains unclear how adolescent exposure to e-cigarette advertising has changed over time and whether exposure to ads might elicit curiosity about e-cigarettes and lead to greater susceptibility to vaping. Most extant studies have looked at only single years in investigating the impact of exposure [11, 27] during a time when companies are adjusting e-cigarette advertising spending and diversifying advertising channels [13, 17]. Examining how exposure to e-cigarette advertising has changed over time and the role exposure plays in impacting adolescent curiosity and susceptibility is of great importance. In fact, further work on this topic could advance an understanding of e-cigarette advertising and adolescent e-cigarette use and provide public health professionals and regulatory agencies valuable insights into how to more effectively regulate e-cigarette advertising to better discourage youth vaping.

We used the National Youth Tobacco Surveys (NYTSs) from 2014 to 2020 to examine: (1) adolescent exposure to e-cigarette advertising over time; and (2) associations between exposure to e-cigarette advertising and both curiosity about e-cigarettes and susceptibility to using e-cigarettes among US adolescents over this period.

## Methods

Data were obtained from the National Youth Tobacco Surveys (NYTSs). The NYTS is a cross-sectional, nationally representative annual survey conducted by the CDC to evaluate the use of tobacco products in U.S. middle and high school students. The NYTS began to include measures related to e-cigarette use in 2014. The 2021 NYTS data may not be comparable with previous NYTS survey waves given a change in survey data collection from in-school to online data collection due to the COVID-19 pandemic [28]. Therefore, the current analysis examined the 2014-2020 NYTS data. This study uses publicly available data, therefore, approval from the Institutional Review Board was waived.

### Participants

The sample for this study included U.S. middle and high school students who reported never having used e-cigarettes, similar to other studies [12, 29]. Survey participants were asked, “Have you ever used an e-cigarette, even once or twice?” Participants who answered “No” to this question were considered never users and were included in this study (N = 97,496), with the percentages that represented never users for each year in 2014-2020 being 78%, 71.4%, 76%, 76.4%, 71.6%, 65.6%, and 74.1%, respectively. The final study participants consisted of 50.3% female and 45.5% non-Hispanic white. See Table 1 for detailed participant information (See Table 2 for weighted percentage).

**Table 1:** Sample demographics—National Youth Tobacco Surveys, 2014-2020.

Year (N, %)							
	2014	2015	2016	2017	2018	2019	2020
Sex							
Male	8494 (49.1%)	6191 (48.6%)	7736 (49.0%)	6674 (48.5%)	7070 (48.5%)	6346 (50.5%)	5294 (49.1%)
Female	8674 (50.2%)	6452 (50.7%)	7981 (50.5%)	6972 (50.7%)	7381 (50.7%)	6132 (48.8%)	5470 (50.7%)
Race							
Non-Hispanic White	7733 (44.7%)	6175 (48.5%)	6770 (42.8%)	6224 (45.3%)	6675 (45.8%)	5863 (46.7%)	4945 (45.8%)
Non-Hispanic Black	2849 (16.5%)	1950 (15.3%)	2662 (16.8%)	2648 (19.3%)	2103 (14.4%)	1786 (14.2%)	1391 (12.9%)
Hispanic	4613 (26.7%)	3218 (25.3%)	4208 (26.6%)	3375 (24.5%)	4205 (28.9%)	3651 (29.1%)	3148 (29.2%)
Non-Hispanic other	1180 (6.8%)	777 (6.1%)	1391 (8.8%)	906 (6.6%)	906 (6.2%)	937 (7.5%)	983 (9.1%)
Grade							
Middle school	9084 (52.6%)	6889 (54.1%)	8307 (52.6%)	6578 (47.8%)	7697 (52.8%)	7052 (56.1%)	6269 (58.1%)
High school	8105 (46.9%)	5790 (45.5%)	7435 (47.1%)	7115 (51.7%)	6791 (46.6%)	5458 (43.4%)	4500 (41.7%)
Other	9 (0.1%)	3 (0.0%)	6 (0.0%)	3 (0.0%)	1 (0.0%)	15 (0.1%)	16 (0.1%)
Exposure: Internet							
No exposure	4903 (28.4%)	3248 (25.5%)	3864 (24.5%)	5500 (40.0%)	5757 (39.5%)	3846 (30.6%)	3259 (30.2%)
Medium exposure	10246 (59.3%)	7848 (61.7%)	9884 (62.6%)	6785 (49.3%)	7116 (48.8%)	6936 (55.2%)	6021 (55.8%)
High exposure	1702 (9.8%)	1311 (10.3%)	1536 (9.7%)	915 (6.7%)	1083 (7.4%)	1620 (12.9%)	1374 (12.7%)
Exposure: Newspapers and magazines							
No exposure	7741 (44.8%)	5686 (44.7%)	8294 (52.5%)	8443 (61.4%)	9600 (65.9%)	8211 (65.4%)	7315 (67.8%)
Medium exposure	7783 (45.0%)	5776 (45.4%)	6070 (38.4%)	4152 (30.2%)	3763 (25.8%)	3487 (27.8%)	2736 (25.3%)
High exposure	1326 (7.7%)	927 (7.3%)	882 (5.6%)	579 (4.2%)	579 (4.0%)	662 (5.3%)	535 (5.0%)
Exposure: Convenience stores							
No exposure	3769 (21.8%)	2319 (18.2%)	2583 (16.3%)	4156 (30.2%)	4644 (31.9%)	2943 (23.4%)	2402 (22.3%)
Medium exposure	8899 (51.5%)	6456 (50.7%)	6648 (42.1%)	6547 (47.6%)	6715 (46.1%)	5995 (47.7%)	4939 (45.8%)
High exposure	4174 (24.1%)	3599 (28.3%)	5946 (37.6%)	2465 (17.9%)	2572 (17.7%)	3416 (27.2%)	3224 (29.9%)
Exposure: TV							
No exposure	5458 (31.6%)	3141 (24.7%)	4524 (28.6%)	5449 (39.6%)	6322 (43.4%)	6335 (50.4%)	5440 (50.4%)
Medium exposure	9489 (54.9%)	7607 (59.8%)	8751 (55.4%)	6794 (49.4%)	6720 (46.1%)	5165 (41.1%)	4394 (40.7%)
High exposure	1876 (10.9%)	1626 (12.8%)	1850 (11.7%)	1030 (7.5%)	1018 (7.0%)	838 (6.7%)	745 (6.9%)

Note: Data were unweighted.

**Table 2:** Demographics (Weighted)—National Youth Tobacco Surveys, 2014-2020.

Weighted %)							
	2014	2015	2016	2017	2018	2019	2020
Sex							
Male	49.0%	49.3%	49.1%	50.2%	49.4%	51.5%	50.2%
Female	51.0%	50.7%	50.9%	49.8%	50.6%	48.5%	49.8%
Race							
Non-Hispanic White	58.0%	57.6%	55.6%	55.5%	53.5%	53.7%	51.0%
Non-Hispanic Black	16.2%	15.2%	14.1%	14.4%	14.6%	15.0%	14.4%
Hispanic	20.9%	22.0%	24.3%	23.7%	25.2%	24.9%	25.7%
Non-Hispanic other	4.9%	5.2%	6.0%	6.4%	6.7%	6.4%	8.9%
Grade							
Middle school	49.2%	52.3%	50.2%	50.0%	52.3%	54.3%	53.3%
High school	50.8%	47.7%	49.8%	50.0%	47.7%	45.6%	46.6%
Other	0.1%	0.0%	0.0%	0.0%	0.0%	0.1%	0.2%
Exposure: Internet							
No exposure	28.3%	26.3%	24.4%	42.4%	40.9%	30.4%	29.7%
Medium exposure	61.8%	63.4%	65.6%	51.4%	51.5%	56.2%	57.7%
High exposure	9.8%	10.3%	9.9%	6.2%	7.6%	13.3%	12.6%
Exposure: Newspapers and magazines							
No exposure	45.2%	45.9%	53.5%	65.2%	68.4%	66.0%	68.2%
Medium exposure	47.1%	46.5%	40.6%	31.1%	27.3%	28.8%	26.7%
High exposure	7.7%	7.6%	5.9%	3.8%	4.3%	5.2%	5.2%
Exposure: Convenience stores							
No exposure	21.8%	19.0%	15.8%	31.3%	33.2%	23.2%	21.6%
Medium exposure	53.4%	51.6%	43.6%	50.5%	48.6%	48.9%	47.4%
High exposure	24.8%	29.4%	40.6%	18.2%	18.3%	27.9%	31.0%
Exposure: TV							
No exposure	31.9%	25.1%	30.1%	42.2%	45.5%	51.1%	50.7%
Medium exposure	57.0%	61.8%	58.1%	51.0%	47.5%	42.0%	42.4%
High exposure	11.1%	13.1%	11.9%	6.8%	7.0%	6.8%	7.0%

Note: Data were weighted.

### Measures

#### E-cigarette advertising exposure

Each year from 2014 to 2020, the NYTS included self-reported exposure to e-cigarette advertising from four channels: Internet, newspapers and magazines, convenience stores, and TV. For each exposure channel, participants were asked, “When you are (using the channel), how often do you see ads or promotions for e-cigarettes?” Participants answered the questions with seven response categories, “I do not (use the channel)”, “Never”, “Rarely”, “Sometimes”, “Most of the time”, or “Always”. Following prior practices, participants were exclusively classified as “no exposure” (“Do not use the channel” and “Never”), “medium exposure” (“Rarely” and “Sometimes”), or “high exposure” (“Most of the time” and “Always”) for each channel [12, 27].

#### Curiosity and susceptibility

Both concepts were measured in our paper in the standard way that has been done in the literature for decades [12, 13, 30]. Curiosity was treated separately from the susceptibility measure. Curiosity was measured by the question, “Have you ever been curious about using an e-cigarette?” [13]. Participants self-reported their curiosity as “Definitely yes”, “Probably yes”, “Probably not”, and “Definitely not”. Participants who reported “Definitely not” were considered “Not curious”, otherwise participants were considered “Curious”. For 2014 and 2015, two questions were used to measure susceptibility: “Do you think that you will try an e-cigarette soon?” and “If one of your best friends were to offer you an e-cigarette, would you use it?” From 2016 through 2020, one more item was added to measure susceptibility, “Do you think you will use an e-cigarette in the next year?” Response categories were the same as that for the curiosity measure. Participants who answered “Definitely not” to these questions were classified as “Not susceptible”, with everyone else being considered “Susceptible” [12, 29].

#### Covariates

Sex, race/ethnicity, and grade levels were included as covariates as they are the most commonly used demographics in prior research on adolescent tobacco product use and were found to be related to e-cigarette advertising exposure, curiosity, and susceptibility among adolescents [9, 11–13, 31, 32]

#### Analytic strategy

Ordinal logistic regressions were used to examine the change in exposure to e-cigarette advertising over time from 2014 to 2020. For exposure from each channel, the three levels of exposure were treated as ordinal. Entered covariates included sex, race/ethnicity, and grade level. General multinomial logistic regression models were fitted to examine the association between exposure and both curiosity and susceptibility. In each model, exposure channel, survey year, exposure channel by survey year interactions as the predictors, and covariables were entered simultaneously, and curiosity or susceptibility as the outcome variable was modeled. For each exposure channel, “no exposure” was treated as the referent, to which “medium exposure” and “high exposure” were compared. Adjusted odds ratios (AORs) and 95% confidence intervals (CIs) were reported. Data were weighted to account for the complex survey design and adjusted for nonresponse. SAS Version 9.4 was used for performing analyses.

## Results

### Exposure to e-cigarette advertising in 2014-2020

**Fig 1:**
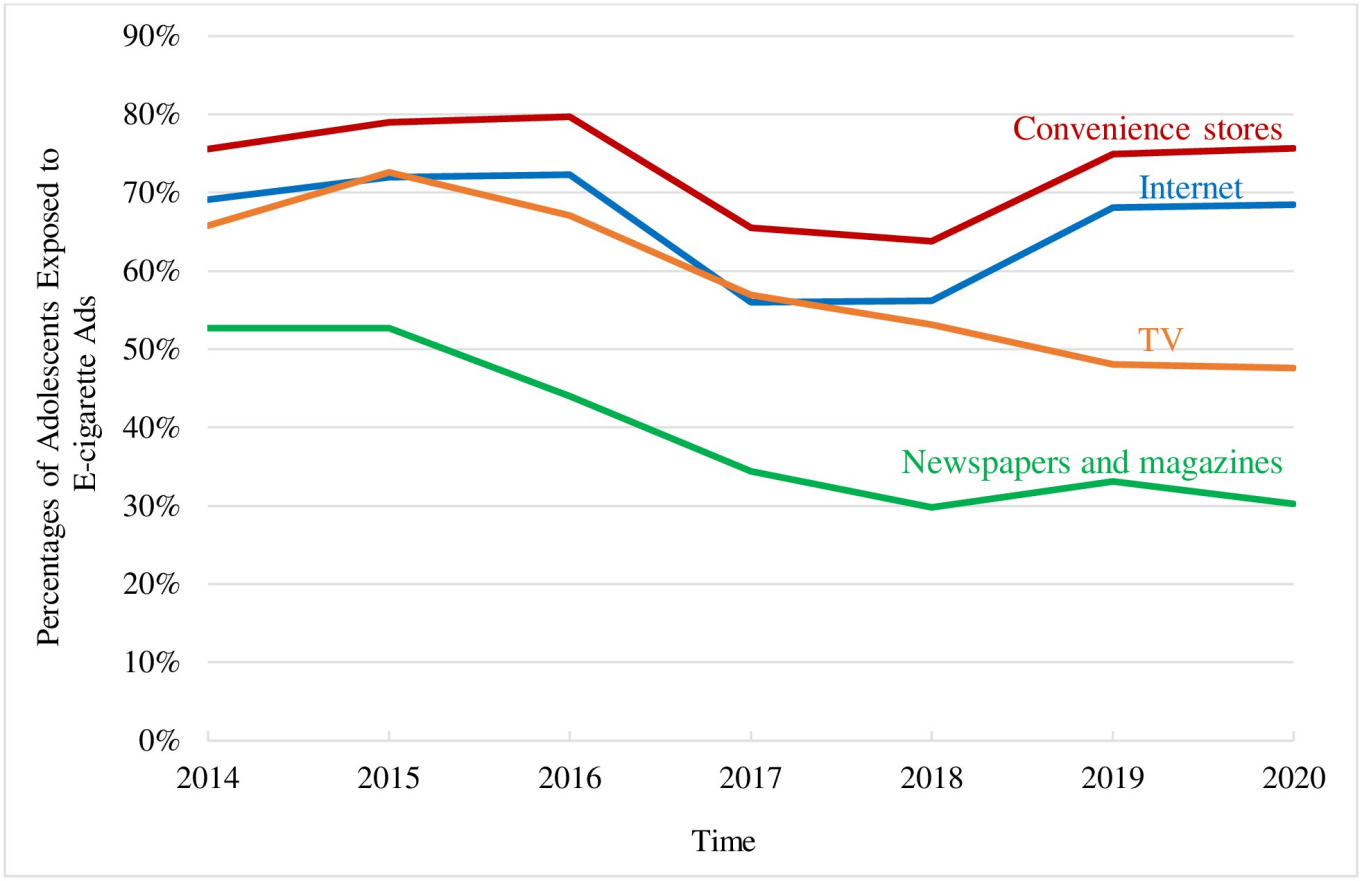
Adolescent exposure to e-cigarette advertising over time—National Youth Tobacco Surveys, 2014-2020.

Across all survey years, convenience stores and the Internet are the two channels for which survey adolescents reported the most e-cigarette advertising exposure on average, with 76.3% (95% CI [76.0%, 76.6%]) and 68.2% (95% CI [67.8%, 68.6%]) of survey adolescents reporting exposure, respectively, followed by TV (60.5%, 95% CI [60.1%, 60.9%]) and newspapers and magazines (41.1%, 95% CI [40.7%, 41.5%]). Exposure to e-cigarette advertising on the Internet and in convenience stores formed an increase-decrease-increase pattern from 2014 to 2020; whereas, exposure in newspapers and magazines and on TV gradually decreased (See Fig 1).

For exposure to e-cigarette advertising on the Internet, the odds that participants reported exposure (vs. no exposure) increased in 2015 (AOR = 1.1, 95% CI [1.0, 1.2]) compared to 2014. Exposure on the Internet did not change in 2016 (AOR = 1.1, 95% CI [0.9, 1.1]), decreased in 2017 (AOR = 0.5, 95% CI [0.4, 0.5]), and increased in 2018 (AOR = 1.1, 95% CI [1.0, 1.2]) and 2019 (AOR = 1.6, 95% CI [1.5, 1.7]). In 2020 (AOR = 1.0, 95% CI [0.9, 1.1]), advertising exposure on the Internet did not change compared to 2019.

Exposure to e-cigarette advertising in newspapers and magazines in 2015 (AOR = 1.0, 95% CI [0.9, 1.1]) did not change compared to 2014. Afterwards, it decreased in 2016 (AOR = 0.7, 95% CI [0.7, 0.8]), 2017 (AOR = 0.6, 95% CI [0.6, 0.7]), and 2018 (AOR = 0.9, 95% CI [0.8, 1.0]). Exposure increased again in 2019 (AOR = 1.1, 95% CI [1.1, 1.2]), followed by a decrease in 2020 (AOR = 0.9, 95% CI [0.9, 1.0]).

Exposure to e-cigarette advertising in convenience stores in 2015 (AOR = 1.3, 95% CI [1.2, 1.4]) increased compared to 2014. Exposure increased again in 2016 (AOR = 1.6, 95% CI [1.4, 1.8]) but decreased in 2017 (AOR = 0.3, 95% CI [0.3, 0.4]) and maintained at a similar level in 2018 (AOR = 0.9, 95% CI [0.9, 1.0]). Afterwards, exposure increased in 2019 (AOR = 1.9, 95% CI [1.7, 2.1]) and 2020 (AOR = 1.2, 95% CI [1.0, 1.3]).

Exposure to e-cigarette advertising on TV increased in 2015 (AOR = 1.3, 95% CI [1.2, 1.5]) compared to 2014. Afterwards, it decreased in 2016 (AOR = 0.8, 95% CI [0.8, 0.9]), 2017 (AOR = 0.6, 95% CI [0.6, 0.7]), 2018 (AOR = 0.9, 95% CI [0.8, 1.0]), and 2019 (AOR = 0.8, 95% CI [0.8, 0.9]). Exposure did not change in 2020 (AOR = 1.0, 95% CI [0.9, 1.1]) (See Table 3).

**Table 3:** Change in exposure to e-cigarette advertising for each year compared to previous year—National Youth Tobacco Surveys, 2014-2020.

	AOR	95%CI	p
Internet			
2015 vs. 2014	1.1	1.0, 1.2	0.038
2016 vs. 2015	1.1	1.0, 1.1	0.162
2017 vs. 2016	0.5	0.5, 0.6	<.001
2018 vs. 2017	1.1	1.0, 1.2	<.001
2019 vs. 2018	1.6	1.5, 1.7	<.001
2020 vs. 2019	1.0	0.9, 1.1	0.999
Newspaper and magazine			
2015 vs. 2014	1.0	0.9, 1.1	0.665
2016 vs. 2015	0.7	0.7, 0.8	<.001
2017 vs. 2016	0.6	0.6, 0.7	<.001
2018 vs. 2017	0.9	0.8, 1.0	<.001
2019 vs. 2018	1.1	1.1, 1.2	0.001
2020 vs. 2019	0.9	0.9, 1.0	0.028
Convenience stores			
2015 vs. 2014	1.3	1.2, 1.4	<.001
2016 vs. 2015	1.6	1.4, 1.8	<.001
2017 vs. 2016	0.3	0.3, 0.3	<.001
2018 vs. 2017	0.9	0.9, 1.0	0.118
2019 vs. 2018	1.9	1.7, 2.1	<.001
2020 vs. 2019	1.2	1.0, 1.3	0.011
TV			
2015 vs. 2014	1.3	1.2, 1.5	<.001
2016 vs. 2015	0.8	0.8, 0.9	<.001
2017 vs. 2016	0.6	0.6, 0.7	<.001
2018 vs. 2017	0.9	0.8, 1.0	0.023
2019 vs. 2018	0.8	0.8, 0.9	<.001
2020 vs. 2019	1.0	0.9, 1.1	0.557

Note: AOR = Adjusted odds ratio. The AORs are adjusted for covariables (i.e., sex, race/ethnicity, and grade level). CI = Confidence Interval. Data were weighted to account for the complex survey design and adjusted for nonresponse.

### Associations between exposure and curiosity and susceptibility

#### Exposure and curiosity

Starting from 2014, participants with “medium exposure” to e-cigarette advertising on the Internet reported being significantly more curious about e-cigarettes compared to the “no exposure” reference group (AOR = 1.6, 95% CI [1.4, 1.8]); so did for the “high exposure” participants (AOR = 1.6, 95% CI [1.3, 2.0]) compared to “no exposure” group. Over the following years, the association between exposure and curiosity remained significant for both “medium exposure” and “high exposure” participants. Participants with “medium exposure” and “high exposure” to e-cigarette advertising in newspapers and magazines were not more curious compared to the “no exposure” group across all survey years except for “medium exposure” participants in 2016 (AOR = 1.1, 95% CI [1.0, 1.2]) and 2017 (AOR = 1.2, 95% CI [1.0, 1.4]), and “high exposure” participants in 2019 (AOR = 0.7, 95% CI [0.5, 0.9]).

Beginning in 2014, compared to “no exposure” participants, participants who reported “medium exposure” (AOR = 1.2, 95% CI [1.0, 1.5] and “high exposure” (AOR = 1.3, 95% CI [1.1, 1.5]) to e-cigarette advertising in convenience stores became more curious about e-cigarettes, except for 2015 for “high exposure” participants (AOR = 1.2, 95% CI [1.0, 1.4]). Participants with “high exposure” to e-cigarette advertising on TV were less curious than “no exposure” participants in 2018 (AOR = 0.8, 95% CI [0.6, 1.0]), 2019 (AOR = 0.8, 95% CI [0.7, 1.0]), and 2020 (AOR = 0.7, 95% CI [0.6, 1.0]) (See Table 4).

**Table 4:** Parameter estimates (AOR and CI) for curiosity by year—National Youth Tobacco Surveys, 2014-2020.

	2014	2015	2016	2017	2018	2019	2020
Internet							
No exposure	Reference	Reference	Reference	Reference	Reference	Reference	Reference
Medium exposure	1.6 (1.4, 1.8)	1.5 (1.3, 1.8)	1.6 (1.4, 1.8)	1.5 (1.3, 1.7)	1.5 (1.3, 1.8)	1.7 (1.5, 1.9)	1.6 (1.4, 1.9)
High exposure	1.6 (1.3, 2.0)	1.4 (1.1, 1.8)	1.8 (1.4, 2.2)	1.39 (1.0, 1.9)	2.0 (1.6, 2.5)	2.1 (1.7, 2.5)	1.9 (1.6, 2.3)
Newspapers and magazines							
No exposure	Reference	Reference	Reference	Reference	Reference	Reference	Reference
Medium exposure	1.0 (0.9, 1.1)	1.1 (0.9, 1.2)	1.1 (1.0, 1.2)	1.2 (1.0, 1.4)	1.0 (0.9, 1.2)	1.0 (0.9, 1.1)	1.0 (0.9, 1.11)
High exposure	1.0 (0.8, 1.1)	1.1 (0.8, 1.4)	1.1 (0.9, 1.4)	1.1 (0.8, 1.5)	0.8 (0.5, 1.1)	0.7 (0.5, 0.9)	1.0 (0.7, 1.3)
Convenience stores							
No exposure	Reference	Reference	Reference	Reference	Reference	Reference	Reference
Medium exposure	1.2 (1.0, 1.5)	1.2 (1.0, 1.3)	1.3 (1.2, 1.5)	1.3 (1.2, 1.6)	1.3 (1.1, 1.5)	1.2 (1.1, 1.4)	1.3 (1.1, 1.5)
High exposure	1.3 (1.1, 1.5)	1.2 (1.0, 1.4)	1.4 (1.2, 1.6)	1.5 (1.3, 1.8)	1.3 (1.1, 1.5)	1.5 (1.3, 1.8)	1.5 (1.3, 1.9)
TV							
No exposure	Reference	Reference	Reference	Reference	Reference	Reference	Reference
Medium exposure	1.1 (1.0, 1.3)	1.0 (1.0, 1.2)	1.1 (1.0, 1.2)	0.9 (0.8, 1.0)	1.0 (0.9, 1.1)	1.0 (0.9, 1.1)	1.1 (1.0, 1.2)
High exposure	0.9 (0.7, 1.1)	0.9 (0.8, 1.1)	0.9 (0.7, 1.1)	0.8 (0.6, 1.1)	0.8 (0.6, 1.0)	0.8 (0.7, 1.0)	0.7 (0.6, 0.9)

Note. AOR = Adjusted odds ratio. The AORs are adjusted for covariables (i.e., sex, race/ethnicity, and grade level). CI = 95% Confidence Interval. Data were weighted to account for the complex survey design and adjusted for nonresponse.

#### Exposure and susceptibility

Beginning in 2014, compared to the “no exposure” reference group, participants with “medium exposure” (AOR = 1.5, 95% CI [1.4, 1.7]) and “high exposure” (AOR = 1.6, 95% CI [1.3, 1.9]) to e-cigarette advertising on the Internet were more susceptible to using e-cigarettes. Over the following years, the associations between “medium exposure” and “high exposure” and susceptibility remained significant. Participants with “medium exposure” and “high exposure” to e-cigarette advertising in newspapers and magazines were not more susceptible to using e-cigarettes across survey years, except for “medium exposure” participants in 2017 (AOR = 1.2, 95% CI [1.1, 1.4]) and “high exposure” participants in 2018 (AOR = 0.7, 95% CI [0.5, 0.9]) and 2019 (AOR = 0.7, 95% CI [0.6, 0.9]).

Starting from 2016, participants with “medium exposure” (AOR = 1.2, 95% CI [1.1, 1.4]) and “high exposure” (AOR = 1.2, 95% CI [1.0, 1.4]) to e-cigarette advertising in convenience stores were more susceptible to using e-cigarettes, compared to the “no exposure” reference group, except for “medium exposure” participants in 2019 (AOR = 1.1, 95% CI [0.9, 1.3]). Participants with “medium exposure” to e-cigarette advertising on TV were more susceptible to using e-cigarettes compared to “no exposure” group, in 2014 (AOR = 1.2, 95% CI [1.0, 1.4]), 2016 (AOR = 1.2, 95% CI [1.1, 1.3]), and 2020 (AOR = 1.2, 95% CI [1.1, 1.3]) (See Table 5).

**Table 5:** Parameter estimates (AOR and CI) for susceptibility by year—National Youth Tobacco Surveys, 2014-2020.

	2014	2015	2016	2017	2018	2019	2020
Internet							
No exposure	Reference	Reference	Reference	Reference	Reference	Reference	Reference
Medium exposure	1.5 (1.4, 1.7)	1.4 (1.2, 1.6)	1.3 (1.1, 1.5)	1.5 (1.3, 1.7)	1.5 (1.3, 1.7)	1.59 (1.4, 1.8)	1.3 (1.2, 1.6)
High exposure	1.6 (1.3, 1.9)	1.3 (1.0, 1.7)	1.6 (1.3, 1.9)	1.5 (1.1, 1.9)	1.9 (1.5, 2.4)	2.0 (1.7, 2.5)	1.6 (1.3, 2.0)
Newspapers and magazines							
No exposure	Reference	Reference	Reference	Reference	Reference	Reference	Reference
Medium exposure	1.0 (1.0, 1.1)	1.1 (0.9, 1.2)	1.1 (1.0, 1.2)	1.2 (1.1, 1.4)	1.0 (0.9, 1.1)	1.0 (0.9, 1.2)	1.1 (1.0, 1.3)
High exposure	1.0 (0.9, 1.2)	1.2 (0.9, 1.5)	0.9 (0.7, 1.2)	1.2 (0.9, 1.5)	0.7 (0.5, 0.9)	0.7 (0.6, 0.9)	0.8 (0.6, 1.1)
Convenience stores							
No exposure	Reference	Reference	Reference	Reference	Reference	Reference	Reference
Medium exposure	1.1 (1.0, 1.3)	1.1 (1.0, 1.3)	1.2 (1.1, 1.4)	1.3 (1.1, 1.5)	1.3 (1.1, 1.5)	1.1 (0.9, 1.3)	1.2 (1.0, 1.4)
High exposure	1.1 (1.0, 1.3)	1.1 (0.9, 1.4)	1.2 (1.0, 1.4)	1.3 (1.0, 1.5)	1.2 (1.1, 1.5)	1.3 (1.1, 1.6)	1.2 (1.0, 1.5)
TV							
No exposure	Reference	Reference	Reference	Reference	Reference	Reference	Reference
Medium exposure	1.2 (1.0, 1.4)	1.1 (1.0, 1.3)	1.2 (1.1, 1.3)	1.0 (0.9, 1.1)	1.0 (0.9, 1.1)	1.0 (0.9, 1.2)	1.2 (1.1, 1.3)
High exposure	1.0 (0.9, 1.2)	1.0 (0.8, 1.2)	1.1 (1.0, 1.3)	1.0 (0.8, 1.3)	0.9 (0.8, 1.2)	1.0 (0.8, 1.1)	1.2 (0.9, 1.5)

Note. AOR = Adjusted odds ratio. The AORs are adjusted for covariables (i.e., sex, race/ethnicity, and grade level). CI = 95% Confidence Interval. Data were weighted to account for the complex survey design and adjusted for nonresponse.

## Discussion

Despite the evolving environment of e-cigarette advertising, products, and regulations [15, 20, 22, 23], little research has examined adolescent exposure to e-cigarette advertising over time and how exposure may relate to antecedents of e-cigarette use across years. The current study explored the pattern of adolescent exposure to e-cigarette advertising from 2014 to 2020 and the associations between exposure and curiosity and susceptibility among U.S. middle and high school students who have never used e-cigarettes, using the National Youth Tobacco Surveys, 2014-2020. Findings of the current study replicate studies examining single year advertising exposure and its impact, and further raise concerns about the potential impact of exposure to e-cigarette advertising on youth uptake of e-cigarettes.

Over time, our results observed different patterns in e-cigarette advertising exposure for multiple channels among adolescents. Exposure to e-cigarette advertising on the Internet and in convenience stores formed an increase-decrease-increase pattern from 2014 to 2020, in which exposure increased from 2014 to 2015, after a decrease in 2016 and 2017, exposure increased again since 2018 around the time that JUUL had gained a leading market share. In contrast, exposure to e-cigarette advertising in newspapers, magazines, and TV gradually decreased over time during survey years. These findings echo some prior work [33] and align with extant NYTS findings showing that e-cigarette advertising exposure was led by the Internet and in-store venues [34]. It could be that adolescents are accessing less print media and TV over time. The finding suggests that adolescents generally see more e-cigarette advertising on the Internet and in convenience stores over time, while seeing less e-cigarette advertising in newspapers and magazines and on TV. The decrease in exposure around 2017 mirrors major tobacco companies’ record low e-cigarette advertising expenditures, due to a lull in TV advertising in 2017, increased advertising by a newer manufactures, and a shift toward social media [17, 35].

Extensive exposure to e-cigarette advertising appears to come at a cost. We found that greater youth exposure to e-cigarette advertising on the Internet and in convenience stores was related to both higher curiosity and higher susceptibility to using e-cigarettes. We found this to be true across multiple years, and our findings align with other single-year studies [11, 36]. For instance, Margolis et al. observed that high (vs. not high) exposure to e-cigarette advertising in stores was associated with greater curiosity [13]. Pu and Zhang similarly observed that exposure to e-cigarette advertising on the Internet and in stores significantly increased the likelihood of adolescents using e-cigarettes [36]. Our study further bolsters these findings, showing that e-cigarette advertising is consistently associated with curiosity and susceptibility across multiple years in national samples of adolescents. The consistent positive associations between exposure and curiosity and susceptibility are especially worrisome, considering that many public campaigns were launched during this time to inform adolescents of the risks of vaping, for instance, the FDA’s The Real Cost campaign [37] and the Truth Initiative’s Safer ≠ Safe campaign [38] in 2018. Continuous efforts are needed to educate adolescents about the risks of vaping to counteract the impact of e-cigarette advertising exposure.

Exposure to e-cigarette advertising in newspapers and magazines and TV was generally not associated with curiosity and susceptibility. One possible explanation was that e-cigarette advertising exposure in newspapers and magazines and TV may not impact e-cigarette risk perception that is shown to be negatively associated with curiosity [9] and susceptibility [39]. Indeed, one study has found that advertising exposure differently impacted e-cigarette harm perception, with advertising exposure via print media or large signs not being related to e-cigarette harm perception [40]. Advertising exposure through TV or radio was not associated with e-cigarette harm perception among e-cigarette never users [40].

Widespread exposure of adolescents to e-cigarette advertising on the Internet and in convenience stores is more disconcerting, as such advertisements may increase the likelihood of experimentation in adolescents for whom advertisements were one of the primary information sources about e-cigarettes [41]. Over 6 million middle school students saw e-cigarette ads at convenience stores in 2014 and over 4 million high school students were exposed to e-cigarette advertising on the Internet [15]. This is not surprising given that 89% of adolescents regularly use the Internet [42] and 77% of schools have at least one tobacco retail outlet within walking distance [43]. With the possibility of increased e-cigarette advertising on the Internet and in retail stores near schools or communities, more adolescents who have never used e-cigarettes could become more curious about e-cigarettes and susceptible to vaping. Therefore, comprehensive youth tobacco regulations should consider restricting e-cigarette advertising in environments that adolescents visit regularly, particularly the Internet and stores, to reduce exposure and corresponding curiosity and susceptibility. On the other hand, the finding regarding the power of Internet exposure also suggests the potential use of the platform in fostering negative attitudes and social norms about e-cigarettes through counter-advertising campaigns. As Pew research showed, 95% of teens have access to a smartphone and 45% of them report they are online “almost constantly” [42]. Taking full advantage of the online environment for e-cigarette prevention campaigns will thus be important to discouraging vaping among youth.

The study is subject to several limitations. First, a bidirectional relationship between exposure to e-cigarette advertising and both curiosity and susceptibility may exist. It is possible that youth who are more curious about and susceptible to e-cigarettes are more likely to notice e-cigarette advertising. Longitudinal studies are needed to tease out a possible bi-directional relationship. Nevertheless, recent longitudinal research has observed a link between advertising exposure and e-cigarette use [33, 44, 45]. Second, respondents may think the Internet includes social media in answering the question about e-cigarette advertising exposure on the Internet, which may impact their responses to some extent. Future research should define the Internet in the survey question and make it clear that the Internet does not include social media to explore the impact of Internet sites only as a channel of e-cigarette exposure. Third, exposure to e-cigarette advertising on channels other than those identified in the NYTS may also be associated with curiosity and susceptibility. For instance, social media may serve as an important channel for e-cigarette advertising exposure, as tobacco companies spend millions in advertising on social media and many adolescents regularly visit social media platforms [41, 46]. There is ample user-generated content promoting vaping on many social media platforms [47]. However, the current analysis was unable to examine how exposure to e-cigarette advertising on social media may influence curiosity and susceptibility over time since social media exposure data were collected only for 2020 in the NYTS. Thus, our study likely underestimates adolescents’ exposure to e-cigarette advertising, given the lack of data on channels such as social media. Fourth, the current study only looked at participants who were exposed to the advertising channels. Future research should also compare the difference between adolescents exposed and not exposed to each channel because there may be unmeasured variables contributing to the relationship. For instance, if some non-exposed youth vape at similar rates as some exposed youth, it may be other influences at play (e.g., social factors). Finally, in our analysis, exposure channel, survey year by itself, and survey year by exposure channel interactions were included as predictors, allowing us to see the exposure change in patterns. Therefore, a trend analysis was not performed. Future research could formally test trends over time.

## Conclusion

In conclusion, the current study showed that adolescents have been consistently exposed to e-cigarette advertising on the Internet and in convenience stores, with decreased exposure to e-cigarette advertising in newspapers, magazines, and on TV over time. We also found that e-cigarette advertising exposure on the Internet and in convenience stores was consistently associated with more curiosity about and susceptibility to use e-cigarettes. These findings suggest the increase in e-cigarette advertising carries significant public health risks for adolescents since exposure to these advertisements could potentially increase curiosity and susceptibility. Therefore, comprehensive tobacco prevention efforts to prevent and reduce e-cigarette use among adolescents should regulate e-cigarette advertising, especially on the Internet and in convenience stores, thereby potentially reducing adolescent exposure to the advertising and ultimately discouraging use [18]. In addition to regulations, continuous efforts are also needed to educate adolescents about the risks of vaping to counteract the impact of e-cigarette advertising exposure. Risks of exposure to e-cigarette advertising would presumably increase as adolescents spend more time online and as convenience stores continue to grow the market share of e-cigarettes [48, 49]. Continued surveillance of e-cigarette advertising exposure and associations with important predictors of use thus should be a priority for tobacco control researchers to more effectively direct e-cigarette advertising regulations and policymaking.
